# *N*-(5-Benzyl­sulfanyl-1,3,4-thia­diazol-2-yl)-2-(piperidin-1-yl)acetamide

**DOI:** 10.1107/S160053681400213X

**Published:** 2014-02-05

**Authors:** D. S. Ismailova, R. Ya. Okmanov, A. A. Ziyaev, Kh. M. Shakhidoyatov, B. Tashkhodjaev

**Affiliations:** a S. Yunusov Institute of the Chemistry of Plant Substances, Academy of Sciences of Uzbekistan, Mirzo Ulugbek Str. 77, Tashkent 100170, Uzbekistan

## Abstract

The title compound, C_16_H_20_N_4_OS_2_, was synthesized by the reaction of 2-benzyl­sulfanyl-5-chloro­acetamido-1,3,4-thia­diazole and piperidine in a 1:2 ratio. The planes of the acetamide and 1,3,4-thia­diazole units are twisted by 10.8 (4)°. The thia­diazole S atom and the acetamide O atom are *syn*-oriented due to a hypervalent S⋯O inter­action of 2.628 (4) Å. In the crystal, mol­ecules form centrosymmetric dimers *via* N—H⋯N hydrogen bonds. These dimers are further connected by C—H⋯O inter­actions into (100) layers.

## Related literature   

For physiological properties and syntheses of 1,3,4-thia­diazole derivatives, see: Turner *et al.* (1988 ▶); Chapleo *et al.* (1987 ▶); Cleici *et al.* (2001 ▶); Jain & Mishra (2004 ▶). For the structures of related 1,3,4-thia­diazole derivatives, see: Leung *et al.* (1992 ▶); Zhang (2009 ▶).
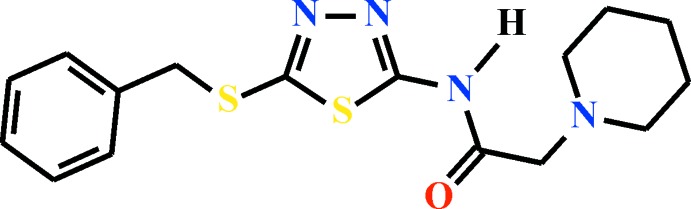


## Experimental   

### 

#### Crystal data  

C_16_H_20_N_4_OS_2_
*M* *_r_* = 348.48
Monoclinic, 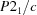

*a* = 17.429 (4) Å
*b* = 16.748 (3) Å
*c* = 5.8390 (12) Å
β = 95.48 (3)°
*V* = 1696.6 (6) Å^3^
*Z* = 4
Cu *K*α radiation
μ = 2.92 mm^−1^
*T* = 290 K
0.35 × 0.28 × 0.20 mm

#### Data collection  

Oxford Diffraction Xcalibur Ruby diffractometer
Absorption correction: multi-scan (*CrysAlis PRO*; Oxford Diffraction, 2009 ▶) *T*_min_ = 0.431, *T*_max_ = 0.558
7003 measured reflections
2970 independent reflections
1521 reflections with *I* > 2σ(*I*)
*R*_int_ = 0.117

#### Refinement  

*R*[*F* ^2^ > 2σ(*F* ^2^)] = 0.069
*wR*(*F* ^2^) = 0.217
*S* = 0.98
2970 reflections
208 parameters
H-atom parameters constrained
Δρ_max_ = 0.41 e Å^−3^
Δρ_min_ = −0.39 e Å^−3^

### 

Data collection: *CrysAlis PRO* (Oxford Diffraction, 2009 ▶); cell refinement: *CrysAlis PRO*; data reduction: *CrysAlis PRO*; program(s) used to solve structure: *SHELXS97* (Sheldrick, 2008 ▶); program(s) used to refine structure: *SHELXS97* (Sheldrick, 2008 ▶); molecular graphics: *XP* in *SHELXTL* (Sheldrick, 2008 ▶); software used to prepare material for publication: *SHELXTL*, *PLATON* (Spek, 2009 ▶) and *publCIF* (Westrip, 2010 ▶).

## Supplementary Material

Crystal structure: contains datablock(s) I, Global. DOI: 10.1107/S160053681400213X/gk2599sup1.cif

Structure factors: contains datablock(s) I. DOI: 10.1107/S160053681400213X/gk2599Isup2.hkl

Supporting information file. DOI: 10.1107/S160053681400213X/gk2599Isup3.cml

CCDC reference:

Additional supporting information:  crystallographic information; 3D view; checkCIF report

## Figures and Tables

**Table 1:** Hydrogen-bond geometry (Å, °)

*D*—H⋯*A*	*D*—H	H⋯*A*	*D*⋯*A*	*D*—H⋯*A*
N3—H3⋯N1^i^	0.86	2.08	2.930 (7)	169
C11—H11*A*⋯O1^ii^	0.97	2.55	3.334 (8)	138

Symmetry codes: (i) 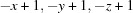; (ii) 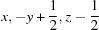.

**Fig. 1.**
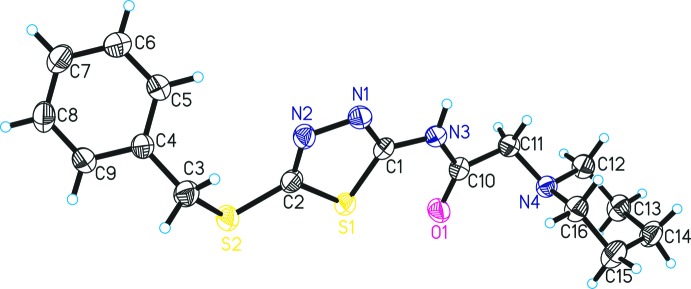
The molecular structure of the title compound. The displacement ellipsoid are drawn at the 30% probability level.

**Fig. 2.**
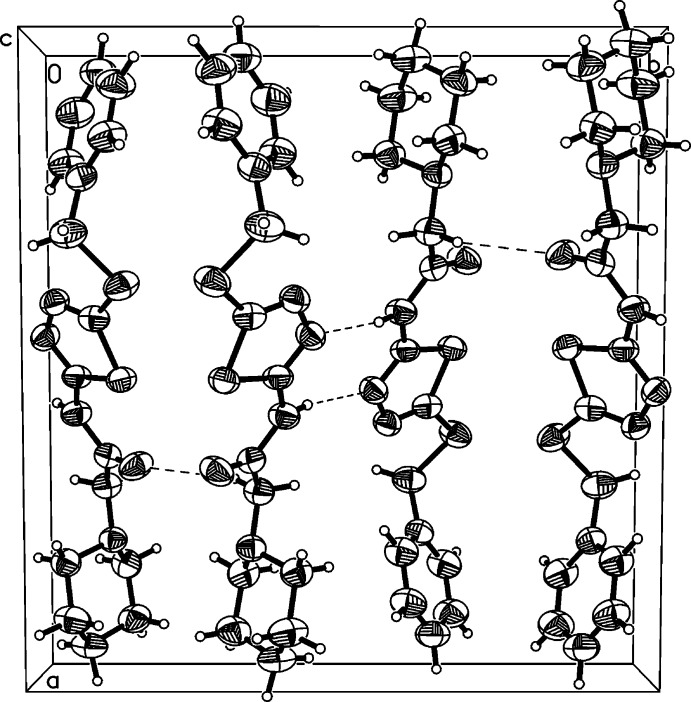
Crystal packing viewed down the c axis with hydrogen bonds shown as dashed lines.

## supplementary crystallographic information

### 1. Comment

1,3,4-Thiadiazoless are a very important class of compounds because of their interesting physiological properties. Derivatives of 1,3,4– thiadiazoles show different biological activities such as antihypertensive (Turner *et al.*, 1988), anticonvulsant (Chapleo *et al.*, 1987), anti-depressant (Cleici *et al.*, 2001), and diuretic (Jain & Mishra, 2004). Acetazolamide, having a 1,3,4-thiadiazole moiety, is known in medicine as narcotic drug.

The title compound was obtained in the reaction of 2-benzylsulfanyl-5-chloroacetamido-1,3,4-thiadiazole and piperidine in a 1:2 ratio in the presence of benzene. The structure of the obtained product was confirmed by single-crystal X-ray analysis and ^1^H NMR spectroscopy.

Molecular structure of title compound is shown in Figure 1. The acetamido-1,3,4-thiadiazole (S1/C1/N1/N2/C2/N3/C10/O1/C11) unit is essentially planar [r.m.s. deviation 0.082 Å]. The thiadiazole sulfur and the acetamido oxygen atoms are *syn* oriented due to a hypervalent interaction with the S···O distance of 2.628 (4) Å. In crystal, the molecules form centrosymmetric dimers through N-H···N hydrogen bonds (Table 1, Fig. 2). These dimers are further connected by C11—H···O1 interactions into (100) layers. As well as an intramolecular S···O hypervalent interaction [S1···O1 ═ 2.625 (5) Å] was observed.

### 2. Experimental

To a solution of 2.99 g (10 mmol) of 2–benzylsulfanyl–5–chloroacetamido–1,3,4–thiadiazole in 15 ml benzene was added dropwise 1.7 g (20 mmol) of piperidine at room temperature. The reaction mixture was refluxed for 8 h. Benzene was distilled off, the residue was washed with water, 2% solution of NaOH, again with water and re-crystallized from hexane [yield 3.02 g (87%); m.p. 376–377 K]. Colourless crystals suitable for *X*–ray analysis were grown from hexane at room temperature.

### 3. Refinement

The H atoms were placed geometrically with N—H ═ 0.86 Å, C—H =0.93 Å for C_ar_ or 0.97 Å for methylene group and included in the refinement in a riding model approximation with *U*_iso_=1.2*U*_eq_(C, N)

### Crystal data

**Table:**

C_16_H_20_N_4_OS_2_	*F*(000) = 736
*M**_r_* = 348.48	*D*_x_ = 1.364 Mg m^−^^3^
Monoclinic, *P*2_1_/*c*	Melting point < 376(1) K
Hall symbol: -P 2ybc	Cu *K*α radiation, λ = 1.54184 Å
*a* = 17.429 (4) Å	Cell parameters from 124 reflections
*b* = 16.748 (3) Å	θ = 5.9–35.8°
*c* = 5.8390 (12) Å	µ = 2.92 mm^−^^1^
β = 95.48 (3)°	*T* = 290 K
*V* = 1696.6 (6) Å^3^	Prizmatic, colourless
*Z* = 4	0.35 × 0.28 × 0.20 mm

### Data collection

**Table:**

Oxford Diffraction Xcalibur Ruby diffractometer	2970 independent reflections
Radiation source: Enhance (Cu) X-ray Source	1521 reflections with *I* > 2σ(*I*)
Graphite monochromator	*R*_int_ = 0.117
Detector resolution: 10.2576 pixels mm^-1^	θ_max_ = 66.6°, θ_min_ = 3.7°
ω scans	*h* = −20→20
Absorption correction: multi-scan (*CrysAlis PRO*; Oxford Diffraction, 2009)	*k* = 0→19
*T*_min_ = 0.431, *T*_max_ = 0.558	*l* = 0→6
7003 measured reflections	

### Refinement

**Table:**

Refinement on *F*^2^	Primary atom site location: structure-invariant direct methods
Least-squares matrix: full	Secondary atom site location: difference Fourier map
*R*[*F*^2^ > 2σ(*F*^2^)] = 0.069	Hydrogen site location: inferred from neighbouring sites
*wR*(*F*^2^) = 0.217	H-atom parameters constrained
*S* = 0.98	*w* = 1/[σ^2^(*F*_o_^2^) + (0.1068*P*)^2^] where *P* = (*F*_o_^2^ + 2*F*_c_^2^)/3
2970 reflections	(Δ/σ)_max_ < 0.001
208 parameters	Δρ_max_ = 0.41 e Å^−^^3^
0 restraints	Δρ_min_ = −0.39 e Å^−^^3^

### Special details

**Table:**

Experimental. ^1^H NMR (400 MHz, CDCl_3_, DMSO): 7.30 (5*H*, m, H–5,6,7,8,9), 6.36 (1*H*, s, N–H), 4.39 (2*H*, s, CH_2_–11), 3.17 (2*H*, s CH_2_–3), 2.47 (4*H*, t, J═5.0 Hz, CH_2_–12,16), 1.57 (4*H*, m, CH_2_–13,15), 1.42 (2*H*, t, J═5.1 Hz, CH_2_–14).
Geometry. All esds (except the esd in the dihedral angle between two l.s. planes) are estimated using the full covariance matrix. The cell esds are taken into account individually in the estimation of esds in distances, angles and torsion angles; correlations between esds in cell parameters are only used when they are defined by crystal symmetry. An approximate (isotropic) treatment of cell esds is used for estimating esds involving l.s. planes.
Refinement. Refinement of *F*^2^ against ALL reflections. The weighted *R*-factor *wR* and goodness of fit *S* are based on *F*^2^, conventional *R*-factors *R* are based on *F*, with *F* set to zero for negative *F*^2^. The threshold expression of *F*^2^ > σ(*F*^2^) is used only for calculating *R*-factors(gt) *etc*. and is not relevant to the choice of reflections for refinement. *R*-factors based on *F*^2^ are statistically about twice as large as those based on *F*, and *R*- factors based on ALL data will be even larger.

### Fractional atomic coordinates and isotropic or equivalent isotropic displacement parameters (Å^2^)

**Table:**

	*x*	*y*	*z*	*U*_iso_*/*U*_eq_	
S1	0.52312 (7)	0.33111 (9)	0.9429 (2)	0.0528 (4)	
S2	0.39119 (9)	0.32063 (11)	1.2305 (3)	0.0642 (5)	
O1	0.6512 (2)	0.3164 (3)	0.7429 (8)	0.0675 (12)	
N1	0.4609 (3)	0.4532 (3)	0.7342 (8)	0.0545 (12)	
N2	0.4113 (3)	0.4313 (3)	0.8972 (8)	0.0536 (12)	
N3	0.5742 (3)	0.4130 (3)	0.5819 (8)	0.0549 (12)	
H3	0.5681	0.4490	0.4769	0.066*	
N4	0.7677 (3)	0.3620 (3)	0.4568 (7)	0.0499 (11)	
C1	0.5195 (3)	0.4055 (4)	0.7371 (9)	0.0496 (14)	
C2	0.4357 (3)	0.3686 (4)	1.0141 (9)	0.0524 (15)	
C3	0.3196 (3)	0.3937 (4)	1.3030 (10)	0.0637 (17)	
H3B	0.3186	0.3945	1.4688	0.076*	
H3C	0.3357	0.4461	1.2561	0.076*	
C4	0.2399 (3)	0.3782 (4)	1.1947 (9)	0.0526 (14)	
C5	0.2152 (3)	0.4084 (4)	0.9797 (10)	0.0641 (17)	
H5A	0.2496	0.4359	0.8964	0.077*	
C6	0.1403 (4)	0.3980 (5)	0.8881 (11)	0.074 (2)	
H6A	0.1248	0.4181	0.7426	0.089*	
C7	0.0882 (4)	0.3586 (4)	1.0077 (12)	0.0730 (19)	
H7A	0.0371	0.3534	0.9472	0.088*	
C8	0.1127 (4)	0.3267 (5)	1.2187 (12)	0.077 (2)	
H8A	0.0782	0.2981	1.2991	0.093*	
C9	0.1871 (3)	0.3364 (4)	1.3124 (11)	0.0677 (18)	
H9A	0.2025	0.3147	1.4562	0.081*	
C10	0.6380 (3)	0.3646 (4)	0.5903 (10)	0.0500 (14)	
C11	0.6864 (3)	0.3734 (4)	0.3892 (9)	0.0550 (15)	
H11A	0.6694	0.3347	0.2717	0.066*	
H11B	0.6784	0.4263	0.3231	0.066*	
C12	0.8104 (3)	0.3476 (4)	0.2561 (10)	0.0606 (16)	
H12A	0.8052	0.3935	0.1545	0.073*	
H12B	0.7888	0.3016	0.1719	0.073*	
C13	0.8937 (3)	0.3331 (4)	0.3287 (11)	0.0691 (18)	
H13A	0.9210	0.3260	0.1930	0.083*	
H13B	0.8988	0.2841	0.4176	0.083*	
C14	0.9299 (3)	0.4006 (5)	0.4705 (11)	0.074 (2)	
H14A	0.9324	0.4477	0.3750	0.089*	
H14B	0.9821	0.3862	0.5281	0.089*	
C15	0.8832 (4)	0.4190 (5)	0.6722 (11)	0.075 (2)	
H15A	0.8879	0.3751	0.7811	0.090*	
H15B	0.9032	0.4667	0.7506	0.090*	
C16	0.7994 (3)	0.4313 (4)	0.5881 (10)	0.0592 (16)	
H16A	0.7701	0.4401	0.7188	0.071*	
H16B	0.7944	0.4784	0.4914	0.071*	

### Atomic displacement parameters (Å^2^)

**Table:**

	*U*^11^	*U*^22^	*U*^33^	*U*^12^	*U*^13^	*U*^23^
S1	0.0408 (7)	0.0584 (9)	0.0596 (9)	0.0043 (7)	0.0063 (6)	0.0094 (8)
S2	0.0531 (8)	0.0744 (11)	0.0673 (10)	0.0131 (8)	0.0177 (7)	0.0167 (9)
O1	0.053 (2)	0.074 (3)	0.077 (3)	0.014 (2)	0.017 (2)	0.030 (3)
N1	0.051 (3)	0.053 (3)	0.059 (3)	0.008 (2)	0.006 (2)	0.007 (2)
N2	0.045 (3)	0.055 (3)	0.062 (3)	0.004 (2)	0.011 (2)	0.008 (3)
N3	0.045 (2)	0.064 (3)	0.055 (3)	0.001 (2)	0.005 (2)	0.014 (3)
N4	0.045 (2)	0.058 (3)	0.047 (3)	0.002 (2)	0.0067 (19)	−0.003 (2)
C1	0.036 (3)	0.058 (4)	0.054 (3)	−0.001 (2)	0.000 (2)	0.003 (3)
C2	0.042 (3)	0.063 (4)	0.052 (3)	−0.001 (3)	0.004 (2)	−0.003 (3)
C3	0.053 (3)	0.085 (5)	0.054 (4)	0.005 (3)	0.008 (3)	−0.007 (3)
C4	0.050 (3)	0.059 (4)	0.050 (3)	0.004 (3)	0.011 (2)	−0.001 (3)
C5	0.057 (4)	0.079 (5)	0.057 (4)	0.000 (3)	0.012 (3)	0.012 (3)
C6	0.065 (4)	0.092 (6)	0.063 (4)	0.006 (4)	−0.001 (3)	0.009 (4)
C7	0.047 (3)	0.085 (5)	0.086 (5)	0.000 (3)	0.003 (3)	−0.002 (4)
C8	0.056 (4)	0.090 (5)	0.089 (5)	−0.006 (4)	0.025 (3)	0.016 (4)
C9	0.059 (4)	0.088 (5)	0.058 (4)	0.007 (3)	0.015 (3)	0.018 (4)
C10	0.040 (3)	0.050 (3)	0.060 (4)	−0.002 (2)	0.005 (2)	−0.002 (3)
C11	0.050 (3)	0.060 (4)	0.055 (3)	0.002 (3)	0.007 (3)	0.003 (3)
C12	0.061 (4)	0.070 (4)	0.052 (3)	−0.002 (3)	0.012 (3)	−0.012 (3)
C13	0.056 (3)	0.084 (5)	0.070 (4)	0.008 (4)	0.022 (3)	−0.011 (4)
C14	0.044 (3)	0.099 (6)	0.078 (5)	−0.003 (3)	0.004 (3)	−0.005 (4)
C15	0.054 (4)	0.102 (6)	0.071 (4)	−0.006 (4)	0.007 (3)	−0.027 (4)
C16	0.054 (3)	0.066 (4)	0.058 (4)	0.001 (3)	0.013 (3)	−0.014 (3)

### Geometric parameters (Å, º)

**Table:**

S1—C1	1.728 (6)	C7—C8	1.373 (9)
S1—C2	1.736 (5)	C7—H7A	0.9300
S2—C2	1.741 (6)	C8—C9	1.368 (9)
S2—C3	1.825 (6)	C8—H8A	0.9300
O1—C10	1.208 (7)	C9—H9A	0.9300
N1—C1	1.295 (7)	C10—C11	1.517 (7)
N1—N2	1.394 (6)	C11—H11A	0.9700
N2—C2	1.301 (8)	C11—H11B	0.9700
N3—C10	1.372 (7)	C12—C13	1.492 (8)
N3—C1	1.383 (7)	C12—H12A	0.9700
N3—H3	0.8600	C12—H12B	0.9700
N4—C11	1.448 (7)	C13—C14	1.504 (9)
N4—C12	1.467 (7)	C13—H13A	0.9700
N4—C16	1.469 (7)	C13—H13B	0.9700
C3—C4	1.493 (8)	C14—C15	1.527 (8)
C3—H3B	0.9700	C14—H14A	0.9700
C3—H3C	0.9700	C14—H14B	0.9700
C4—C5	1.383 (8)	C15—C16	1.509 (8)
C4—C9	1.390 (8)	C15—H15A	0.9700
C5—C6	1.373 (8)	C15—H15B	0.9700
C5—H5A	0.9300	C16—H16A	0.9700
C6—C7	1.368 (9)	C16—H16B	0.9700
C6—H6A	0.9300		
			
C1—S1—C2	86.0 (3)	O1—C10—N3	121.2 (5)
C2—S2—C3	102.7 (3)	O1—C10—C11	123.7 (5)
C1—N1—N2	111.6 (5)	N3—C10—C11	115.0 (5)
C2—N2—N1	112.3 (4)	N4—C11—C10	112.2 (5)
C10—N3—C1	122.1 (5)	N4—C11—H11A	109.2
C10—N3—H3	119.0	C10—C11—H11A	109.2
C1—N3—H3	119.0	N4—C11—H11B	109.2
C11—N4—C12	111.3 (4)	C10—C11—H11B	109.2
C11—N4—C16	110.3 (5)	H11A—C11—H11B	107.9
C12—N4—C16	110.6 (5)	N4—C12—C13	110.7 (5)
N1—C1—N3	121.9 (5)	N4—C12—H12A	109.5
N1—C1—S1	115.5 (4)	C13—C12—H12A	109.5
N3—C1—S1	122.6 (4)	N4—C12—H12B	109.5
N2—C2—S1	114.6 (4)	C13—C12—H12B	109.5
N2—C2—S2	127.4 (4)	H12A—C12—H12B	108.1
S1—C2—S2	118.0 (4)	C12—C13—C14	112.3 (5)
C4—C3—S2	114.5 (5)	C12—C13—H13A	109.1
C4—C3—H3B	108.6	C14—C13—H13A	109.1
S2—C3—H3B	108.6	C12—C13—H13B	109.1
C4—C3—H3C	108.6	C14—C13—H13B	109.1
S2—C3—H3C	108.6	H13A—C13—H13B	107.9
H3B—C3—H3C	107.6	C13—C14—C15	110.4 (5)
C5—C4—C9	118.0 (6)	C13—C14—H14A	109.6
C5—C4—C3	121.2 (5)	C15—C14—H14A	109.6
C9—C4—C3	120.7 (6)	C13—C14—H14B	109.6
C6—C5—C4	120.6 (6)	C15—C14—H14B	109.6
C6—C5—H5A	119.7	H14A—C14—H14B	108.1
C4—C5—H5A	119.7	C16—C15—C14	110.3 (5)
C7—C6—C5	121.0 (7)	C16—C15—H15A	109.6
C7—C6—H6A	119.5	C14—C15—H15A	109.6
C5—C6—H6A	119.5	C16—C15—H15B	109.6
C6—C7—C8	118.7 (6)	C14—C15—H15B	109.6
C6—C7—H7A	120.6	H15A—C15—H15B	108.1
C8—C7—H7A	120.6	N4—C16—C15	111.5 (5)
C9—C8—C7	121.0 (6)	N4—C16—H16A	109.3
C9—C8—H8A	119.5	C15—C16—H16A	109.3
C7—C8—H8A	119.5	N4—C16—H16B	109.3
C8—C9—C4	120.6 (6)	C15—C16—H16B	109.3
C8—C9—H9A	119.7	H16A—C16—H16B	108.0
C4—C9—H9A	119.7		
			
C1—N1—N2—C2	0.3 (7)	C5—C6—C7—C8	−2.4 (12)
N2—N1—C1—N3	176.5 (5)	C6—C7—C8—C9	2.3 (12)
N2—N1—C1—S1	−2.1 (7)	C7—C8—C9—C4	−0.6 (12)
C10—N3—C1—N1	177.3 (5)	C5—C4—C9—C8	−1.1 (10)
C10—N3—C1—S1	−4.2 (8)	C3—C4—C9—C8	175.8 (6)
C2—S1—C1—N1	2.5 (5)	C1—N3—C10—O1	−4.4 (9)
C2—S1—C1—N3	−176.1 (5)	C1—N3—C10—C11	172.2 (5)
N1—N2—C2—S1	1.7 (7)	C12—N4—C11—C10	163.5 (5)
N1—N2—C2—S2	−179.1 (4)	C16—N4—C11—C10	−73.3 (6)
C1—S1—C2—N2	−2.3 (5)	O1—C10—C11—N4	−37.3 (8)
C1—S1—C2—S2	178.4 (4)	N3—C10—C11—N4	146.2 (5)
C3—S2—C2—N2	−14.8 (6)	C11—N4—C12—C13	−178.0 (5)
C3—S2—C2—S1	164.4 (3)	C16—N4—C12—C13	58.9 (7)
C2—S2—C3—C4	99.2 (5)	N4—C12—C13—C14	−56.4 (7)
S2—C3—C4—C5	−88.5 (7)	C12—C13—C14—C15	52.9 (8)
S2—C3—C4—C9	94.7 (6)	C13—C14—C15—C16	−52.0 (8)
C9—C4—C5—C6	1.0 (10)	C11—N4—C16—C15	176.8 (5)
C3—C4—C5—C6	−175.9 (6)	C12—N4—C16—C15	−59.6 (7)
C4—C5—C6—C7	0.7 (12)	C14—C15—C16—N4	56.1 (8)

### Hydrogen-bond geometry (Å, º)

**Table:**

*D*—H···*A*	*D*—H	H···*A*	*D*···*A*	*D*—H···*A*
N3—H3···N1^i^	0.86	2.08	2.930 (7)	169
C11—H11*A*···O1^ii^	0.97	2.55	3.334 (8)	138

Symmetry codes: (i) −*x*+1, −*y*+1, −*z*+1; (ii) *x*, −*y*+1/2, *z*−1/2.

## References

[bb1] Chapleo, B. C., Myers, P. L., Smith, A. C. B., Tulloch, I. F. & Walter, D. S. (1987). *J. Med. Chem.* **30**, 5, 951–954. 10.1021/jm00388a038 3572984

[bb2] Cleici, F., Pocar, D., Guido, M., Loche, A., Perlini, V. & Brufani, M. (2001). *J. Med. Chem.* **44**, 931–936. 10.1021/jm001027w 11300875

[bb3] Jain, S. K. & Mishra, P. (2004). *Indian J. Chem. Sect. B*, **43**, 184–188.

[bb4] Leung, B. K.-O., Hudson, M. J. & Drew, M. G. B. (1992). *Transition Met. Chem.* **17**, 352–355.

[bb5] Oxford Diffraction (2009). *CrysAlis PRO.* Oxford Diffraction Ltd, Yarnton, England.

[bb6] Sheldrick, G. M. (2008). *Acta Cryst.* A**64**, 112–122. 10.1107/S0108767307043930 18156677

[bb7] Spek, A. L. (2009). *Acta Cryst.* D**65**, 148–155. 10.1107/S090744490804362X PMC2631630 19171970

[bb8] Turner, S., Myers, M., Gadie, B., Nelson, A. J., Pape, R., Saville, J. F., Doxey, J. C. & Berridge, T. L. (1988). *J. Med. Chem.* **31**, 5, 902–906. 10.1021/jm00400a003 3361578

[bb9] Westrip, S. P. (2010). *J. Appl. Cryst.* **43**, 920–925.

[bb10] Zhang, G.-Y. (2009). *Acta Cryst.* E**65**, o2138. 10.1107/S1600536809030554 PMC2969935 21577548

